# Genotoxic and antigenotoxic properties of selenium compounds in the *in vitro* micronucleus assay with human whole blood lymphocytes and tk6 lymphoblastoid cells

**DOI:** 10.1100/tsw.2006.204

**Published:** 2006-09-25

**Authors:** Eduard Cemeli, Ricard Marcos, Diana Anderson

**Affiliations:** ^2^ Grup de Mutagénesis, Department de Genética I de Microbiologia, Campus de Bellaterra, 08193, Cerdanyola del Valles, Spain; ^1^ Department of Biomedical Sciences, University of Bradford, Bradford, BD7 1DP, West Yorkshire, UK

**Keywords:** sodium selenite, sodium selenate, selenous acid, potassium dichromate, micronucleus, antigenotoxicity, genotoxicity

## Abstract

Selenium is known to possess both genotoxic and antigenotoxic properties. In the present study, we have evaluated the genotoxicity and antigenotoxicity of three selenium compounds (sodium selenate, sodium selenite and selenous acid) by measuring in vitro micronucleus induction. Assays were conducted in whole blood lymphocytes and in the TK6 lymphoblastoid cell line, with and without co-treatment with potassium dichromate, a known genotoxic compound. In general, the compounds were more active in TK6 cells than they were in blood lymphocytes. Only 1 μM selenous acid increased the frequency of binucleated cells containing micronuclei (BNMN) in blood lymphocytes, while all three selenium compounds increased BNMN in TK6 cells. In addition, combinations of selenous acid and potassium dichromate resulted in lower frequencies of BNMN than potassium dichromate alone in blood lymphocytes, while combinations of sodium selenate and potassium dichromate produced lower frequencies of BNMN than potassium dichromate alone in TK6 cells. The concentrations of selenium compounds that were used, in combination with the medium components and the biological physiology of the whole blood lymphocytes and TK6 cells, could have affected the redox potential of the compounds, switching the chemicals from a pro-oxidant to antioxidant status and vice-versa. The lower activities of the compounds in blood lymphocytes may be due to the protective effects of blood components. The results indicate that the genotoxic and antigenotoxic properties of selenium compounds are highly dependent upon the conditions under which they are evaluated.

## INTRODUCTION

Since the toxicity of selenium was first described in 1933 in the U.S.[1], there has been controversy regarding the genotoxic and antigenotoxic properties of this metal. Over the last 2 decades, molecular, clinical, and epidemiological studies have reported that selenium exhibits anticarcinogenic effects[2,3,4]. In contrast, other studies have shown that selenium is genotoxic[1,5,6,7]. Selenium is also essential for nutrition and a wide range of biological functions, and the fact that selenium is ubiquitous in the environment raises concern about its properties.

Concentrations of selenium in plasma and whole blood provide useful indicators of human selenium intake and status. The estimated adequacy level is of 100 µg/l[8]. However, the profound influence of the natural environment on the selenium content of soils, crops, and diets leads to large variability among populations. The mean reference value of selenium in plasma from healthy individuals in the metropolitan area of Barcelona (Spain) obtained was 82.2 ± 17.5 µg/l[9], whereas in Upper Silesia (Poland), it was 62.5 ± 18.4 µg/l, which is below the lower limit of the nutritional adequacy range [80 µg/l][10]. Three communities of Greenland provided a range of concentrations of selenium in plasma from 79 µg/l in Tasiilaq women to 113 µg/l in Uummannaq men[11]. The largest survey to date of selenium status of Australians found a mean plasma concentration of 103 µg/l[12]. The toxicity of selenium depends on the nature of the selenium compound, particularly its solubility. Long-term selenosis was apparent in five patients with blood selenium concentrations in excess of 1.054–1.854 mg/l. This concentration was estimated to represent a selenium intake of 0.91 mg/day. However, this value is arguable since the reduction of intake reverses the effects of selenosis[13].

Chromium, which is extensively absorbed by all routes in humans, is clearly genotoxic[6,14], carcinogenic, and mutagenic in animals and in exposed workers[15]. It is known to participate in the formation of OH• *in vitro*, and OH• may be involved in its *in vivo* toxicity[15], through the generation of lipid peroxidation and DNA damage[16].

Several studies have investigated whether selenium compounds diminish the toxic effects of chromium compounds. The genotoxic activity of three selenium compounds in the somatic cells of *Drosophila* was evaluated and it was found that sodium selenite displayed some activity in reducing the potent genotoxicity of potassium dichromate[17]. The effects of several selenium compounds on the mutagenicity of potassium dichromate in the Ames test and its DNA-damaging ability in human lymphocytes (comet assay) was investigated[18]. It was observed that sodium selenate reduced the genotoxic effects of potassium dichromate in both assays, whereas sodium selenite and selenous acid did not. This study also demonstrated that sodium selenite had no effect on the genetic damage induced by chromium in TK6 human lymphoblastoid cells[18].

Other studies have examined the effects of chromium and selenium on cytogenetic endpoints. It was found that selenous acid suppressed micronucleus induction by potassium dichromate in mice and suggested that the mechanism for the suppression involved metallothionein in mouse bone marrow[16]. The kinetochore staining in the cytokinesis-blocked micronucleus assay was used to demonstrate that chromium salts did not act by damaging these structures[19]. However, they did not investigate the effects of selenium on the types of micronuclei induced by chromium. In the present study, we have evaluated further the effects on selenium on micronucleus induction by chromium. We have studied whether three selenium compounds (sodium selenite, sodium selenate, and selenous acid) affected the cytotoxicity and genotoxicity of potassium dichromate by measuring micronucleus induction in whole human blood lymphocytes and in the TK6 lymphoblastoid cell line.

## MATERIALS AND METHODS

### Materials

Sodium selenite [CAS 10102-18-8; 99%], sodium selenate [CAS 13410-01-0; 98%], and selenous acid [CAS 7783-00-8; 98%] were purchased from Aldrich (Milwakee, WI). Potassium dichromate [CAS 778-

50-9], mytomicin C (MMC), fungicide, penicillin-streptomycin, and cytochalasin-B were from Sigma (Steinheim, Germany). RPMI 1640 with 25 m*M* Hepes, foetal bovine serum, phytohaemagglutinin, and 200 m*M* L-glutamine were purchased from Gibco (Paisley, U.K.). Dimethyl sulfoxide (DMSO), potassium chloride [CAS 7447-40-7], and methanol were obtained from Panreac (Barcelona, Spain). Giemsa's stain solution Gurr®, sodium orthophosphate [CAS 7601-54-9], potassium orthophosphate [CAS 7778-53-2], slides, and coverslips were purchased from BDH (Poole, U.K.), while heparin was from Rovi (Barcelona, Spain) and Histomount was purchased from Fisher (Loughborough, U.K.). Cell culture flasks were from Corning (Corning, NY) and 15-ml tubes were from Greiner bio-one (Stonehouse, U.K.).

## Methods

- **Lymphocyte cell culture and treatment** — Lymphocyte cell cultures were initiated by adding 0.5 ml of heparinised whole blood from a non-smoking, 27-year-old donor to 4.5 ml of 82% RPMI 1640 medium supplemented with 15% foetal calf serum, 1% penicillin-streptomycin, 1% L-glutamine, and 1% phytohaemagglutinin, and incubating the cultures at 37°C in a humidified atmosphere containing 5% $CO_2$. Stock solutions of the test chemicals and the positive control (MMC, at a final concentration of 0.3 μ*M*) were prepared in water and added to the cultures after 24 h of incubation. The volume of chemicals added was always 1% of the volume of the cell culture media. At 44 h from the start of the culture, cytochalasin-B was added at a final concentration of 6 μg/ml to arrest cytokinesis. Cultures were harvested after a total of 72 h of incubation.
- **TK6 cell culture** — TK6 cells were cultured in a medium composed of 87% RPMI 1640 with 25 m*M* Hepes, 10% foetal calf serum, 1% glutamate, 1% penicillin-streptomycin, and 1% fungicide. Three-ml cell cultures were initiated at a concentration of $0.5 \times 10^6$ cells/ml. The test chemicals (at a final volume of 1%), MMC (at a final concentration of 0.2 μ*M*), and cytochalasin-B (at a final concentration of 6 μg/ml) were added immediately. Cell cultures were incubated for 48 h at 37ºC.
- **Slide preparation** — Cells were harvested by centrifugation at $800 \times g$ for 8 min and washed in the appropriate growth medium. The cells were then subjected to a mild "hypotonic" treatment (75 m*M* KCl for 2–3 min at 4ºC), pelleted by centrifugation, and fixed in Carnoy's fixative (methanol: acetic acid, 3:1). Centrifugation followed by fixation was carried out three times. Finally, the fixed cells were resuspended in 80 μl of fixative, and two 20-μl aliquots dropped on each of two slides. The slides were stained with 10% Giemsa® for 10 min and left to dry overnight.
- **Micronucleus analysis** — Slides were coded and scored attending established criteria[20]. One thousand binucleated (BN) cells with well-preserved cytoplasm were scored for each treatment concentration. Micronucleus frequency was expressed as the number of binucleated cells containing micronuclei (BNMN). Five hundred lymphocytes were scored to evaluate the percentage of cells with 1, 2, 3, and 4 or more nuclei. A nuclear division index (cytochalasin-B proliferation index, CBPI) was calculated and the percentage of binucleated cells (%BN) was calculated from the 500 cells scored to obtain the CBPI[20].

All studies were performed on two separate occasions. Differences in CBPI were evaluated using the chi-square test, while Fisher's exact test was used for the BNMN data. Differences of $p < 0.05$ were considered significant.

## RESULTS

Tables 1–4 show the %BN, CBPI, and BNMN data derived from the experiments. Tables 1 and 2 show responses with blood lymphocytes and Tables 3 and 4 data for TK6 lymphoblastoid cells. Dose responses were generated for individual test compound in each cell type. Two concentrations of each compound were selected for the final assays (Tables 1–4) from preliminary range-finding studies with each cell type, one concentration that yielded slight, but significant decreases in CBPI and one with a greater decrease in CBPI that also yielded, where possible, significant increases in the frequency of BNMN. Cytotoxicity was understood to be a 50% decrease in CBPI relative to the negative control[21] and doses with this magnitude of toxicity were not used for the combination treatments.

**TABLE 1**
**Assays Conducted with 75 μ*M* Potassium Dichromate and Selenium Compounds in Whole Blood**

| | BN (%) | CBPI | BNMN |
|---|---|---|---|
| Negative control | 60.8 | 2.15 | 5 |
| 75 μ*M* potassium dichromate | 46.4 | 1.54*** | 12 |
| 5 μ*M* selenous acid | Toxic | Toxic | Toxic |
| 0.5 μ*M* selenous acid | 50.2 | 1.92** | 5 |
| 1 μ*M* sodium selenite | 44.8 | 1.8*** | 7 |
| 0.1 μ*M* sodium selenite | 47.8 | 1.78*** | 5 |
| 50 μ*M* sodium selenate | 50.2 | 1.84** | 4 |
| 5 μ*M* sodium selenate | 60.8 | 2.06*** | 4 |
| Positive control (Mytomicin C) | 49.6 | 1.69*** | 28*** |
| 75 μ*M* potassium dichromate | 46.4 | 1.54 | 12 |
| 75 μ*M* potassium dichromate + 5 μ*M* selenous acid | Toxic | Toxic | Toxic |
| 75 μ*M* potassium dichromate + 0.5 μ*M* selenous acid | 48.2 | 1.63*** | 8 |
| 75 μ*M* potassium dichromate + 1 μ*M* sodium selenite | 48 | 1.63* | 19 |
| 75 μ*M* potassium dichromate + 0.1 μ*M* sodium selenite | 46.4 | 1.58 | 3* |
| 75 μ*M* potassium dichromate + 50 μ*M* sodium selenate | 49.6 | 1.62* | 13 |
| 75 μ*M* potassium dichromate + 5 μ*M* sodium selenate | 52.6 | 1.73*** | 14 |

BN (%), CBPI, BNMN in whole blood treated with 75 μ*M* potassium dichromate, selenous acid, sodium selenite, sodium selenate, and combinations of 75 μ*M* potassium dichromate and selenous acid, sodium selenite, and sodium selenate. *$p < 0.05$, **$p < 0.01$, ***$p < 0.001$.

Combinations of 75 μ*M* potassium dichromate and the selenium compounds were used in one set of tests conducted with whole blood (Table 1). By itself, 75 μ*M* potassium dichromate did not induce a statistically significant increase in MN, but significantly decreased the CBPI. Also, by themselves, the concentrations of the selenium compounds did not generate a significant increase in MN, but they significantly reduced the CBPI. Unfortunately, 5 μ*M* selenous acid resulted in toxicity by itself and in combination with 75 μ*M* potassium dichromate, and thus yielded no usable data. Only the combination of 75 μ*M* potassium dichromate with 0.1 μ*M* sodium selenite significantly reduced the frequency MN produced by potassium dichromate alone. In addition, this was the only combination of 75 μ*M* potassium dichromate and the selenium compounds that did not significantly increase the CBPI produced by 75 μ*M* potassium dichromate alone.

**TABLE 2**
**Assays Conducted with 100 µ*M* Potassium Dichromate and Selenium Compounds in Whole Blood**

| | BN (%) | CBPI | BNMN |
|---|---|---|---|
| Negative control | 52.2 | 1.88 | 4 |
| 100 µ*M* potassium dichromate | 27 | 1.31*** | 22*** |
| 1 µ*M* selenous acid | 60.8 | 2.04*** | 12* |
| Positive control (Mytomicin C) | 55 | 1 | 39*** |
| 100 µ*M* potassium dichromate | 27 | 1.31 | 22 |
| 100 µ*M* potassium dichromate + 1 µ*M* selenous acid | Toxic | Toxic | Toxic |
| 100 µ*M* potassium dichromate + 0.1 µ*M* selenous acid | 27 | 1.31 | 10* |
| 100 µ*M* potassium dichromate + 1 µ*M* sodium selenite | 39.8 | 1.44*** | 13 |
| 100 µ*M* potassium dichromate + 0.1 µ*M* sodium selenite | 29.2 | 1.34 | 18 |
| 100 µ*M* potassium dichromate + 50 µ*M* sodium selenate | 32.4 | 1.37 | 16 |
| 100 µ*M* potassium dichromate + 5 µ*M* sodium selenate | 27.8 | 1.31 | 14 |

BN (%), CBPI, BNMN in whole blood treated with 100 µ*M* potassium dichromate and selenous acid and combinations of 100 µ*M* potassium dichromate, selenous acid, sodium selenite, and sodium selenate. **p* < 0.05, ***p* < 0.01, ****p* < 0.001.

Table 2 shows the results of an additional set of assays conducted in whole blood cultures using 100 µ*M* potassium dichromate, which, by itself, produced significant increases in the frequency of MN as well as significant reductions in CBPI. Also in these assays, selenous acid was tested at a concentration of 1 µ*M*, which by itself increased both CBPI and BNMN in a statistically significant manner. When combinations of 100 µ*M* potassium dichromate and the selenium compounds were tested, all the selenium compounds reduced the frequency of BNMN produced by 100 µ*M* potassium dichromate alone, however, only the combination with 0.1 µ*M* selenous acid reduced BNMN significantly. The combination of 100 µ*M* potassium dichromate and 1 µ*M* selenous acid resulted in excessive toxicity. Only the combination of 100 µ*M* potassium dichromate and 1 µ*M* sodium selenite produced a CBPI that was significantly different from that produced by 100 µ*M* potassium dichromate alone, and the difference was an increase.

Ten n*M* potassium dichromate did not significantly increase the frequency of BNMN in TK6 lymphoblastoid cells; however, this concentration of potassium dichromate produced a slight, but significant decrease of the CBPI (Table 3). All selenium compounds by themselves significantly increased the frequency of BNMN in TK6 cells. The higher concentrations of the selenium compounds significantly decreased the CBPI, while the lower doses had variable effects. None of the combinations of 10 n*M* potassium dichromate and the selenium compounds reduced the frequency of BNMN. In contrast with the hypothesis that the combination results in a reduction, an additive effect was found. Therefore, the CBPI and BNMN frequencies produced by the combination of 10 n*M* potassium dichromate and the selenium compounds were compared to the negative control. All the combinations with the higher concentrations of the selenium compounds induced a statistically significant increase in the frequency of BNMN.

Treatment of TK6 cells with 25 n*M* potassium dichromate produced a significant increase in the frequency of BNMN and a significant reduction in the CBPI (Table 4). When combinations of 25 n*M* potassium dichromate and the selenium compounds were assayed, combinations with sodium selenate resulted in BNMN frequencies that were not different from the negative control, but combinations with selenous acid and sodium selenite resulted in significantly increased BNMN frequencies. The BNMN produced by the combination of 25 n*M* potassium dichromate and 100 µ*M* sodium selenate was also significantly less that the BNMN frequency produced by 25 n*M* potassium dichromate alone.

**TABLE 3**
**Assays Conducted with 10 n*M* Potassium Dichromate and Selenium Compounds in TK6 Lymphoblastoid Cells**

| | BN (%) | CBPI | BNMN |
|---|---|---|---|
| Negative control | 26.8 | 2.38 | 14 |
| 10 n*M* potassium dichromate | 22.2 | 1.92*** | 20 |
| 10 µ*M* selenous acid | 51 | 1.51*** | 11 |
| 1 µ*M* selenous acid | 34.6 | 2.43* | 39*** |
| 10 µ*M* sodium selenite | 43 | 2.01*** | 21 |
| 1 µ*M* sodium selenite | 21.8 | 1.96*** | 49*** |
| 100 µ*M* sodium selenate | 30 | 2.26** | 41*** |
| 10 µ*M* sodium selenate | 25.2 | 2.39 | 18 |
| Positive control (Mytomicin C) | 67.6 | 1.77*** | 74*** |
| 10 n*M* potassium dichromate + 10 µ*M* selenous acid | 51.8 | 1.96*** | 15 |
| 10 n*M* potassium dichromate + 1 µ*M* selenous acid | 31.2 | 2.28** | 27* |
| 10 n*M* potassium dichromate + 10 µ*M* sodium selenite | 53 | 2.12** | 10 |
| 10 n*M* potassium dichromate + 1 µ*M* sodium selenite | 22.6 | 1.79*** | 71*** |
| 10 n*M* potassium dichromate + 100 µ*M* sodium selenate | 21 | 2.24** | 42*** |
| 10 n*M* potassium dichromate + 10 µ*M* sodium selenate | 24.4 | 2.27** | 27* |

BN (%), CBPI, BNMN in TK6 cells treated with 10 n*M* potassium dichromate, selenous acid, sodium selenite, sodium selenate, and combinations of 10 n*M* potassium dichromate and selenous acid, sodium selenite, and sodium selenate. *$p < 0.05$, **$p < 0.01$, ***$p < 0.001$.

## DISCUSSION

The lower concentrations of potassium dichromate used in this study resulted in consistent, but relatively small, increases in the frequency of BNMN in whole blood lymphocytes (Table 1) and TK6 lymphoblastoid cells (Table 2); however, the increases were significant only at the higher concentrations (Tables 2 and 4). These results confirm previous reports on the ability of chromium to generate MN[22,23,24]. A possible mechanism for the genotoxicity of chromium is through the generation of free radicals either by a typical Fenton reaction[25,26] or by another mechanism[27,28].

The mechanisms underlying the various genotoxic and antigenotoxic effects of selenium, however, remain controversial. In terms of clastogenicity and aneugenicity, selenium alone has been reported both to be an inducer of MN and sister chromatid exchange (SCE)[29,30] as well as having no effects[31,32]. In cogenotoxicity assays, it has both enhanced the induction of MN[31] and reduced the formation of DNA damage[33,34,35]. One study indicated that the effects of selenium compounds are dose and time dependent[36]. We also reported that the effects of selenium are highly concentration dependent[18]. In the current investigation, the treatment time for a particular cell type was fixed; however, different concentrations of each selenium compound were used. The higher concentrations that were used approached the maximum level of toxicity acceptable for the assay, whereas the lower concentrations produced relatively low levels of toxicity.

**TABLE 4**
**Assays Conducted with 25 n*M* Potassium Dichromate and Selenium Compounds in TK6 Lymphoblastoid Cells**

| | BN (%) | CBPI | BNMN |
|---|---|---|---|
| Negative control | 36.8 | 2.18 | 11 |
| 25 n*M* potassium dichromate | 32.6 | 2.06** | 24* |
| Positive control (Mytomicin C) | 42 | 1.52*** | 93*** |
| 25 n*M* potassium dichromate | 32.6 | 2.06 | 24 |
| 25 n*M* potassium dichromate + 100 µ*M* sodium selenate | 35.8 | 1.98** | 12* |
| 25 n*M* potassium dichromate + 10 µ*M* sodium selenate | 38.8 | 2.06 | 14 |
| 25 n*M* potassium dichromate + 10 µ*M* selenous acid | 36.2 | 2.02** | 15 |
| 25 n*M* potassium dichromate + 1 µ*M* selenous acid | 36.6 | 2.03** | 32** |
| 25 n*M* potassium dichromate + 10 µ*M* sodium selenite | 34.6 | 2.00** | 42*** |
| 25 n*M* potassium dichromate + 1 µ*M* sodium selenite | 40 | 1.83*** | 25* |
| 25 n*M* potassium dichromate + 100 µ*M* sodium selenate | 35.8 | 1.98*** | 12 |
| 25 n*M* potassium dichromate + 10 µ*M* sodium selenate | 38.8 | 2.06** | 14 |

BN (%), CBPI, BNMN in TK6 cells treated with 25 n*M* potassium dichromate and combinations of 25 n*M* potassium dichromate and selenous acid, sodium selenite, and sodium selenate. $*p < 0.05$, $**p < 0.01$, $***p < 0.001$.

The assays indicated that higher concentrations of selenium compounds by themselves can induce BNMN, especially in TK6 cells. The antigenotoxicity assays that evaluated selenium compounds in combination with potassium dichromate, however, produced rather inconsistent results. The higher dose of potassium dichromate that was assayed generated statistically significant frequencies of BNMN (Tables 2 and 4) in order to evaluate the antigenotoxic effects of the selenium compounds. The frequency of BNMN produced by potassium dichromate was reduced by 100 µ*M* sodium selenate in the TK6 cells and 0.1 µ*M* selenous acid (and possibly sodium selenite) in whole blood. However, cotreatments with potassium dichromate and selenium compounds also resulted in additive effects, particularly in the TK6 cell line. The lack of a consistent pattern of antigenotoxicity or additive genotoxicity might be due to the concentrations of selenium and potassium dichromate selected, in combination with the components of the medium and the biological physiology of the whole blood lymphocytes and the TK6 lymphoblastoid cell line. These factors could have affected the redox potential of the compounds, switching the chemicals from a pro-oxidant status to an antioxidant one or vice versa[37].

Our observations also indicate that TK6 cells were more sensitive than whole blood lymphocytes to the toxicity and genotoxicity of the both the selenium compounds and potassium dichromate. A nanomolar concentration of potassium dichromate induced MN in TK6 cells, while micromolar concentrations were required in whole blood lymphocytes. Although the concentrations of selenium compounds were only slightly higher in assays conducted with TK6 cells than whole blood lymphocytes, the BNMN frequencies and CBPIs were much more affected by the compounds in TK6 cells. The lower sensitivity of whole blood lymphocytes may be explained by the protective effects of blood, which is able to quench a variety of insults[38]. Normal human plasma contains high and low molecular mass redox-active molecules, such as transferrin and caeruloplasmin, that offer considerable protection against organic and inorganic oxygen radicals generated by ions and ascorbate[39]. Caeruloplasmin catalyses the oxidation of ferrous ions to the ferric state (ferroxidase activity), inhibiting lipid peroxidation and the

Fenton reaction[40,41]. Furthermore, the higher sensitivity of TK6 cells towards the chemicals may be due to defective DNA repair systems inherent in tumorigenic cells[42].

Four independent experiments combining potassium dichromate and selenium compounds based on preliminary dose responses for each compound used in this investigation (data not shown) were carried out. This study followed a stepwise progression with the purpose to investigate responses to different concentrations in the combinations in whole blood and in TK6 cells. For each cell type studied, one experiment was performed with a concentration of potassium dichromate providing a small increase in BNMN and another experiment with a concentration of potassium dichromate yielding a statistically significantly increase in BNMN. Since the aim of the study was to investigate the modulation in the generation or suppression of micronucleus by selenium compounds, no evaluation of the morphology of the cells in order to assess apoptosis/necrosis was necessary.

In conclusion, the results of this study measuring BNMN induction in human cells indicate that selenium compounds can be both genotoxic and antigenotoxic. However, the genotoxic and antigenotoxic properties of selenium compounds are highly dependent on the conditions under which the assays are conducted.

*In vivo* experiments, with the same chemicals, in the mouse or rat bone marrow with the micronucleus test may improve the findings of this study.

## ACKNOWLEDGEMENTS

Dr. E. Cemeli was in possession of a scholarship provided by the School of Life Sciences at the University of Bradford when this investigation was carried out.
